# Evaluation of the Role of Dentures & Dietary Advice on Nutritional Status of Complete Edentulous Patients Using MNA®-SF: An Observational Study

**DOI:** 10.7759/cureus.47736

**Published:** 2023-10-26

**Authors:** Sakshi Agrawal, Seema Sathe, Priyanka Paul, Khushbu Doshi, Aastha Agrawal, Nishant Rathi

**Affiliations:** 1 Public Health Dentistry, Sharad Pawar Dental College and Hospital, Datta Meghe Institute of Higher Education and Research (Deemed to be University), Wardha, IND; 2 Prosthodontics, Sharad Pawar Dental College and Hospital, Datta Meghe Institute of Higher Education and Research (Deemed to be University), Wardha, IND

**Keywords:** mastication, teeth, nutritional status, dietary advice, completely edentulous

## Abstract

Aim

This study aims to evaluate the role of dentures and dietary advice on the nutritional status of complete edentulous patients using the mini nutritional assessment-short form (MNA®-SF).

Objective

The objective of this study is to assess patients’ nutrition using MNA®-SF before denture insertion and nutritional advice, to evaluate patients’ nutrition using MNA®-SF after denture insertion and nutritional advice at one month and three months, and to assess and compare pre- and post-insertion differences in nutrition using MNA®-SF.

Material and Method

An observational study was conducted among 50 completely edentulous patients using the MNA®-SF questionnaire. They were asked the questions at baseline and then after they were given complete dentures, after which they were recalled after one month and three months to assess the nutritional status using MNA®-SF. All the questions were in the native language, so it was easy to understand the question and respond to it.

Result

It was found that the patient’s nutritional status had significant changes, which shows that the dietary advice and complete denture affected the patient’s nutritional status.

Conclusions

This study helped assess the efficacy and application of MNA®-SF in completely edentulous patients. In this study, we provided nutritional guidance to patients in addition to complete denture prostheses to encourage them to have healthy eating habits.

## Introduction

The primary function of teeth is mastication, and if teeth are absent, the efficiency of mastication will be hampered, leading to changes in eating habits and raising the likelihood of developing systemic disorders [1,2]. As a result of the loss of teeth that will cause impaired mastication, the patients adapt to that or start swallowing gritty particles that will cause digestive problems [3]. In addition to difficulty in chewing, other factors such as taste and smell changes [4], gastrointestinal decline [5], and depression occur [6]. Impaired nutrient intake, including protein; calcium; iron; vitamins B1, B6, and C; and retinol and beta-carotene, may be related to the development of anorexia in older adults [7,8]. As a result, their diet is deficient in proteins, fibers, and vitamins that can be obtained from raw fruits. The complete denture prosthesis improves the chewing ability as well as health. Hence, it is essential to check their nutritional status occasionally. The mini nutritional assessment-short form (MNA®-SF) is one method of non-invasive investigation before denture insertion.

## Materials and methods

Study setting and design

An observational study was conducted among the complete edentulous patients in the Department of Prosthodontics and Crown & Bridge, Datta Meghe Institute of Higher Education and Research (DMIHER), Wardha, India. The study lasted four months, from April 2023 to July 2023.

Study population

Fifty completely edentulous patients or patients in need of complete dentures were included in the study. The patients were taken from OPD and were first-time denture wearers.

The inclusion criteria are as follows:

1. Patients in need of both upper and lower dentures.

2. Patients who were able to comprehend the clinician’s explanations and instructions.

3. Patients who were able to comprehend and respond to the questionnaire.

4. Patients who were able to follow the dietary instructions.

The exclusion criteria are as follows:

1. A contagious disease.

2. An orofacial motor disorder.

3. Any special dietary requirements or restrictions.

Data collection

The nutritional status of the patients was evaluated using MNA®-SF. The MNA®-SF test is a quick assessment that can be completed in about five minutes. It includes six questions and easy measurements, such as body mass index (BMI) and mobility. The questionnaire also covers dietary habits, food intake, neuropsychological issues, and acute diseases. Based on the score, MNA®-SF indicates three levels of malnutrition: 0-7 for malnutrition, 8-11 for risk of malnutrition, and 12-14 for no malnutrition. The form used for the study was in the local language (Marathi). Dietary advice was provided to patients during their complete denture treatment, considering the different nutritional requirements of males and females. Initially, individuals were categorized as either malnourished, at risk of malnutrition, or well-nourished. Then, they were given complete dentures, and dietary counseling was also done along with the denture. The patients were asked to follow the dietary instructions and return after one month, and then after one month, they were assessed again using MNA®-SF. After three months, the patients were evaluated for their nutritional status. The dietary recommendations were given accordingly. As males’ and females’ nutritional values varied, their dietary needs were distinct and addressed. All conventional denture procedures were followed, and the patients were given initial dietary guidance both during trial insertion and again when the prosthesis was supplied, and dietary advice was given in the documented form. A follow-up call was made for the patients after one month and three months of denture insertion, and MNA®-SF and BMI were analyzed again.

Ethical consideration

After thoroughly explaining the study’s concept and purpose, each participant provided written informed consent. After obtaining ethical approval from the Institutional Ethical Committee (Ref. No. DMIHER(DU)/IEC/2023/702) Sharad Pawar Dental College and Hospital, DMIHER.

Statistical analysis

The data collected were analyzed using IBM SPSS Statistics, version 24.0 (IBM Corp., Armonk, NY). Data for the study were collected using MNA®-SF. Analysis of variance (ANOVA) test was used to determine the relation between the baseline, one-month, and three-month values. ANOVA was used for statistical analysis as three groups were compared simultaneously, as we compared the baseline, one-month, and three-month groups.

## Results

We utilized the ANOVA formula to compare the proportions. The statistical significance in the test above was deemed acceptable based on the p-value of less than 0.05. Table 1 shows the minimum and maximum scores at the baseline, one month, and three months. The minimum and maximum scores at baseline are 6 and 11, respectively. At one month, the minimum and maximum scores are 6 and 11, respectively, and at three months, the minimum and maximum scores are 7 and 12, respectively. These data show that the minimum and maximum values have significant changes.

**Table 1:** Maximum and minimum total scores for the MNA®-SF questionnaire MNA®-SF, mini nutritional assessment-short form

Groups	Minimum score	Maximum score
Baseline	6	11
One month	6	11
Three months	7	12

The data in Table 2 show the average values for the patients’ scores at the beginning of the study, after one month, and after three months. Additionally, it displays the patients’ total scores at the study’s start. In the initial survey, the average score for 50 patients was 8.54. After one month, the average score for 48 patients increased slightly to 8.5417, and after three months, the average score for 46 patients rose to 9.2826. The p-value after comparing the three groups was 0.0331, which is <0.05, indicating that it is significant.

**Table 2:** Statistical values for the study

Groups	No. of patients	Mean	Standard deviation	F statistics	p-value
Baseline	50	8.54	1.5414	3.493	0.0331
one month	48	8.5417	1.5839		
Three months	46	9.2826	1.5869		

Table 3 shows the comparative values and percentage of the people at risk of nutrition and well-nourished. At baseline, 32% (16) were at risk of food, and the rest, 68% (34), were well-nourished. At one month, 25% (12) were at risk of nutrition, and the rest, 75% (36), were well-nourished. At three months, 17.3% (8) were at risk of malnutrition, and 82.6% (38) were well-nourished.

**Table 3:** Comparative values and percentage of the people at risk of nutrition and well-nourished at baseline, one month, and three months

Groups	Risk of malnutrition		Well-nourished		Total	
	Value	Percentage	Value	Percentage	Value	Percentage
Baseline	16	32%	34	68%	50	100%
One month	12	25%	36	75%	48	100%
Three months	8	17.3%	38	82.6%	46	100%
Total	36	25%	108	75%	144	100%

## Discussion

The nutritional status of the patients depends upon several factors, such as physiological, pathological, neurological, psychological, systemic health, oral health, and many more. Patients’ oral health depends upon the saliva, teeth, and mucosa for the proper nutritional intake. The patients’ oral health should be good so that patients’ dietary intake can fulfill the body’s requirements. Having teeth is crucial for proper mastication. Without teeth, one may experience difficulty chewing food, resulting in digestive issues, inadequate nutrient intake, and malnutrition. The act of chewing is the initial stage of digestion, as it enables the food to be adequately prepared for swallowing and further processing in the digestive system [9]. As you chew, the food is broken down into smaller pieces, and your mouth produces saliva to moisten the food and enhance its taste. The occlusal area is where the food is broken down into smaller pieces by the teeth. The level of fragmentation is determined by the overall occlusal surface area, which is in turn influenced by the number of teeth present; if the teeth are absent, there will be an inadequate occlusal surface area that will cause improper fragmentation, and hence, it will lead to improper deglutition and affect the digestion. The usual course of action for individuals without teeth is to receive complete dentures, which aid in restoring their ability to chew. Various studies have demonstrated the impact of the fabrication of complete dentures on nutrient consumption alone. Research shows that complete dentures could enhance nutrient intake [10]. For the dentate status of the patient, the complete denture was given. The complete denture alone cannot satisfy the needs of the patients. It has been found in certain studies that simple dietary advice is necessary to improve the nutritional status of a patient who solely relies on a complete denture [11].

Harris et al. [12] described that the dental environment has been suggested as a suitable place to conduct dietary evaluations and offer advice on nutritional habits. Studies indicate that patients’ dietary behavior can be modified through one-on-one counseling and dental care. Tables 4, 5 show the one-day meal plan for women and men, respectively. Complete dentures alone will improve the masticatory function. Still, along with it, dietary advice will affect the nutritional status of the patient and the elderly, and nutritional intake tends to be inadequate [13]. Therefore, it is crucial to consider protein and micronutrient intake reductions while preventing various diseases like sarcopenia, cardiovascular disease, and osteoporosis [11]. Depending on the individual, edentulousness and prosthetic rehabilitation can affect nutritional status differently. A few studies suggest that dentures can improve the diet quality and food choices of older edentulous individuals undergoing prosthetic rehabilitation [14-16]. However, certain researchers have demonstrated that the use of dentures in rehabilitation did not improve the eating habits of elderly individuals [17]. In this study, the patients needing complete dentures were selected, and based on their requirements and needs, they were assessed for their nutritional status. They were grouped as malnourished at the risk of malnutrition and well-nourished at baseline. Evaluation of retention, stability, and support of the denture after insertion by dental personnel was performed. After the denture insertion, the patient was given post-denture instructions, including diet instructions. During the denture insertion appointment, each patient was directed regarding the proper use of dentures. The importance of follow-up was adequately explained to patients, and the patients were also explained about the denture adaptation related to speech and mastication. The patients were recalled after 24 hours, then after three days, seven days, fifteen days, one month, and three months. For eight to 10 days, patients were asked not to wear dentures during mastication and to practice speaking. They were informed about the excess salivation and comfortability in the initial days. Other than this, the patients were on telephonic follow-up regarding the diet or any issues. For the initial days, when the patients started mastication, they were instructed to have a soft diet and chew bilaterally. Then, the patients were asked to follow the given diet, continuing to masticate the food by bilateral mastication. A few patients were having difficulty adapting dentures. For those patients, alternate-day follow-up was carried out for 10 days, and after satisfactory follow-up, they were given diet instructions. There were few changes in the nutritional assessment at one month, but after three months, there was a significant change in the nutritional evaluation. There was a substantial increase in the consumption of fruits and vegetables compared to the baseline study in the further follow-up. Other than this, the patients became aware of their routine habits and nutritional status and started taking care of their diet consumption. The result showed statistically significant changes in the baseline study and three-month follow-up. This indicates that the dietary advice and complete denture affect the patient’s nutritional status.

**Table 4:** One-day meal plan for women

Time	Diet	Measurement
Early morning, 6.00 am	Tea/coffee/milk	1 cup
Morning 8.00 am	Fruit/lemon water (with sugar and salt)	
Breakfast 9.00 am	Bread/uttapam/upma/mix atta roti/dosa/roti + vegetables/shevai/dhokla/bread sandwich/biscuit	1 plate (large)
Lunch 12.00 pm	Chapati, dal, rice, veg sabaji, curd/kadi/buttermilk, salad, sprouted gram	2/3, 1 cup, 1 cup, 1 cup, 1/2 cup, 1 cup, ½ cup
Snack 4.00 pm	Tea/coffee	1 cup
Dinner 7.00 pm	Same as lunch	

**Table 5:** One-day meal plan for men

Time	Diet	Measurement
Early morning, 6.00 am	Tea/coffee/milk	1 cup
Morning 8.00 am	Fruit/lemon water (with sugar and salt)	
Breakfast 9.00 am	Bread/uttapam/upma/mix atta roti/dosa/roti+vegetabes/shevai/dhokla/bread sandwich/biscuit	One plate
Lunch 12.00 pm	Chapati, dal, rice, veg sabaji, curd/kadi/buttermilk, salad, sprouted gram	4, 1 cup, 1 cup, 1 cup, 1/2 cup, 1 cup, ½ cup
Snack 4.00 pm	Tea/coffee	1 cup
Dinner 7.00 pm	Same as lunch	

Limitations

Other parameters of assessment of nutritional status can be considered. Only oral manifestations are considered for this study. The nutritional deficiency is not solely due to oral manifestation; other factors are also responsible. It’s important to understand that nutritional deficiency can have multiple underlying causes. Among these causes, dentate status (or oral health) is just one of four factors that can impact the dietary selection and overall nutritional status of elderly individuals. The other factors include general health, socioeconomic status, dietary habits, and psychological health. It’s crucial to address these factors when assessing and addressing nutritional deficiencies in older adults.

## Conclusions

If accompanied by dietary advice, rehabilitating completely edentulous patients is an excellent solution for edentulism. There is a positive correlation between complete denture rehabilitation with dietary advice and nutritional status, which is proven in our study. Other than the basic questions, a few questions, such as neuropsychological problems or any stress to the patient, are also responsible for the patient’s nutritional status. This shows that stress is also a factor responsible for the patient’s low dietary intake. This indicates that oral, systemic, and general health should be good; only the patients can improve their nutritional status. Patients also became aware of the diet and nutritional counseling in this study. Prosthetic rehabilitation has improved patients’ ability to chew their food, increasing their intake.
